# Thermal Infrared Tracking Method Based on Efficient Global Information Perception

**DOI:** 10.3390/s22197408

**Published:** 2022-09-29

**Authors:** Long Zhao, Xiaoye Liu, Honge Ren, Lingjixuan Xue

**Affiliations:** 1 Big Data Institute, East University of Heilongjiang, Harbin 150066, China; 2 College of Information and Computer Engineering, Northeast Forestry University, Harbin 150040, China; 3 Forestry Intelligent Equipment Engineering Research Center, Harbin 150040, China

**Keywords:** object tracking, Thermal InfraRed, Transformer

## Abstract

To solve the insufficient ability of the current Thermal InfraRed (TIR) tracking methods to resist occlusion and interference from similar targets, we propose a TIR tracking method based on efficient global information perception. In order to efficiently obtain the global semantic information of images, we use the Transformer structure for feature extraction and fusion. In the feature extraction process, the Focal Transformer structure is used to improve the efficiency of remote information modeling, which is highly similar to the human attention mechanism. The feature fusion process supplements the relative position encoding to the standard Transformer structure, which allows the model to continuously consider the influence of positional relationships during the learning process. It can also generalize to capture the different positional information for different input sequences. Thus, it makes the Transformer structure model the semantic information contained in images more efficiently. To further improve the tracking accuracy and robustness, the heterogeneous bi-prediction head is utilized in the object prediction process. The fully connected sub-network is responsible for the classification prediction of the foreground or background. The convolutional sub-network is responsible for the regression prediction of the object bounding box. In order to alleviate the contradiction between the vast demand for training data of the Transformer model and the insufficient scale of the TIR tracking dataset, the LaSOT-TIR dataset is generated with the generative adversarial network for network training. Our method achieves the best performance compared with other state-of-the-art trackers on the VOT2015-TIR, VOT2017-TIR, PTB-TIR and LSOTB-TIR datasets, and performs outstandingly especially when dealing with severe occlusion or interference from similar objects.

## 1. Introduction

**Figure 1:**
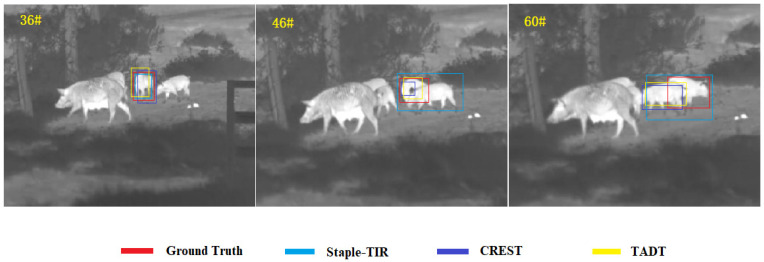
The performance of current methods in dealing with the challenge of interference from similar objects.

In extreme visual environments, such as dark night, heavy fog, rainy and snowy situations, visible light cameras cannot generate images clearly. TIR cameras are insensitive to lighting conditions and have strong penetration, which can capture the infrared radiation with a wavelength of 0.75–13 μm of objects whose temperatures are above absolute zero and form a single-channel grayscale image with high quality [1]. Since TIR target tracking technology can be widely applied in military, industry and manufacture, the tracking methods based on TIR images have been paid more and more attention by scientific researchers in recent years. Most of the current TIR target tracking methods utilize target appearance modeling. These methods try to extract accurate and robust features on the targets in the template frame, and then use the sliding window to match in the specific searching area of the subsequent frame. The specific area of the image that has the highest similarity with the template feature is considered as the area where the target is located. TIR images are sensitive to temperature, prone to thermal crossovers and have no color or texture information. In the open environment, the similarity of the thermal radiation information of objects with the same class in the same location at the same time is extremely high. If the trajectories of the target and the interferer of the same type overlap again at that time (especially the target is blocked by the interferer within a certain period of time), the current TIR tracking method avoids the above challenges with difficulty, and it very easily drifts to the interferer. As shown in Figure 1, this is the tracking result of the current tracker with excellent performance on a video sequence of the TIR target tracking dataset LSOTB-TIR [2]. The red box is the real annotation of the target, Staple-TIR [3] is a TIR target tracking framework with hand-crafted features, and CREST [4] as well as TADT [5] are TIR target tracking frameworks with deep features. The difference between CREST and TADT is that CREST uses the correlation filtering method and TADT is based on the Siamese network. As is shown in Figure 1, Staple-TIR, CREST and TADT can track the target well at the 36-th frame. However, at the 46-th frame, the target is occluded by the interferer and thermally crossovers with the interferer, and the tracking accuracy of the above three trackers begin to drop, especially Staple-TIR with handcrafted features. At the 60-th frame, all three above trackers drift to the interferer. This shows that, although the deep features are more robust than handcrafted features, it is difficult for the trackers to resist the challenges in the above extreme conditions in the TIR target tracking task, no matter whether handcrafted features or deep features are used to represent the target.

We designed the new TIR target tracking method, and it does not use the well-known CNN network in either the feature extraction process or the feature fusion process. It utilizes the latest Transformer [6] network. Transformer’s self-attention mechanism has a strong global perception ability from the beginning of modeling, which solves the limitations of traditional CNN-based or RNN-based models. At the same time, the parallel computing efficiency of Transformer is much higher than that of RNN, and the flexibility is also better than CNN. Transformer focuses on the relative relationship between features rather than the true value of the feature itself. Since Transformer does not completely depend on the training data, the absolute value of the data is no longer as important as in CNN, so it has better generalization and resistance, and can better utilize the synthetic data to train the neural network. However, it also brings challenges due to quadratic computational overhead, especially for the high-resolution vision tasks (e.g., object tracking). Inspired by Focal Transformer [7], our proposed target tracking framework uses the focal self-attention mechanism. This new mechanism can efficiently and effectively capture both short-range and long-range visual dependencies while significantly reducing the computation cost. Our TIR target tracking framework significantly outperforms existing methods on mainstream evaluation datasets.

In this work, we make the following contributions:

- To the best of our knowledge, we are the first to use the Transformer structure in the TIR target tracking method. In the feature extraction process, in order to balance the contradiction between global perception ability and computational complexity, the A Simple and Strong Baseline for Transformer Tracking structure is utilized for feature extraction. The feature fusion process supplements the relative position encoding to the standard Transformer structure, which allows the model to continuously consider the influence of positional relationships during the learning process, and can generalize to capture the different positional information for different input sequences, so that the Transformer structure can more efficiently model the semantic information contained in images.
- The target prediction process creatively utilizes the heterogeneous bi-prediction heads. The fully connected sub-network is responsible for the classification prediction of the foreground or background, and the convolutional sub-network is responsible for the regression prediction of the target bounding box. The experiments show that the heterogeneous prediction heads can further improve the performance of the tracker.
- In order to alleviate the contradiction between the vast demand for training data of the Transformer model and the insufficient scale of the TIR target tracking dataset, the LaSOT-TIR dataset is generated based on the visible light target tracking dataset LaSOT with a generative adversarial network. After using the LaSOT-TIR dataset, the performance of the tracker on the long-term tracking task of the TIR modal targets improves significantly, and we also improve the generalization ability.

## 2. Related Work

Initially, researchers have utilized hand-designed features, such as using one or several methods from raw gray values, oriented gradient histograms and covariance region descriptors, to represent target features. TIR target tracking methods based on particle filter [8] or mean shift [9,10] use these handcrafted features. However, the handcrafted features are not robust enough, limiting the performance of these trackers in complex scenes. In the open environment, the light changes greatly, and there exist many occlusions. It is more difficult for the handcrafted features to accurately express the real state of the target. In this case, no matter what tracking strategy is used, as long as it is based on manual features, both the accuracy and the robustness of the trackers would inevitably drop significantly. With the continuous improvement of deep learning techniques and the TIR target tracking dataset, researchers have begun to use deep features. Compared with handcrafted features, deep features are completely extracted by neural networks, which can contain not only some intuitive visible information but also abstract information in higher levels. In general, the information contained in deep features is more abundant, more discriminative and more independent. Deep features are less affected by common challenges, such as object deformation, illumination changes and occlusion in object tracking tasks. Some studies directly apply the original tracking framework based on visible light targets to TIR targets, such as ECO [11]. The feature extraction networks used in most TIR target tracking frameworks are the same as those used in visible light target tracking frameworks, and they are pre-trained on ImageNet, a large-scale visible light image classification dataset. The same is true for MCFTS [12], which firstly utilizes a pre-trained feature extraction backbone network to extract multiple convolutional layer features of TIR targets, and then utilizes correlation filters to construct multiple weak trackers with corresponding convolutional layer features. These weak trackers provide a response map of the target location. Finally, MCFTS proposes an ensemble method to merge these response maps into a stronger response map. In addition, MCFTS also proposes a simple and effective scale estimation strategy to improve the tracking accuracy. However, the performance of MCFTS is limited by the deep features learned from visible light images for pre-training, and it has poor performance in accurate representation of TIR tracking objects. To solve the above problems, MMNet [13] proposes a multi-task-driven TIR feature model, which simultaneously learns discriminative features and fine-grained correlation features specific for TIR. This method achieves better performance than previous methods on TIR tracking tasks. At the same time, some studies have also considered that images in the TIR modal not only lose color information, but also has insufficient contour information. It is difficult to achieve the ideal tracking accuracy and robustness of trackers only relying on spatial information, so researchers have begun to consider utilizing temporal information. Recurrent neural networks, such as LSTM [14], are widely approved in temporal sequence information processing, so the Ref. [15] combines the advantages of CNN and LSTM for TIR target tracking. Li et al. [16] proposed a TIR object tracking framework using sparse representation of deep semantic features. This deep semantic feature is obtained by a pre-trained VGGNet combined with a target feature channel selection module based on supervised training. Wu et al. [17] proposed a TIR UAV object tracking method based on full convolutional regression network. Zulkifley et al. [18] proposed a TIR object tracking method that combines a binary fully convolutional network with an offline pre-trained Siamese network.

Although the above methods have promoted the development of TIR target tracking research from different aspects, these methods have not solved the problems of interference from similar objects and occlusion in the target tracking task. The above methods only try to strengthen the discrimination of the tracker on the target or design a more powerful appearance representation model to suppress the interferer, but ignore the usage of the information of the interferer itself, which does not model the relationship between the target and the similar objects and makes it difficult to deal with the occlusion problem. Due to the lack of feature information including color and texture features, the feature information of TIR targets is less abundant than that of visible light targets. If the TIR target tracking framework is designed following the previous idea of simply calculating similarity, it is more difficult to deal with challenges such as interference from similar objects and the occlusion situation.

## 3. Methods

**Figure 2:**
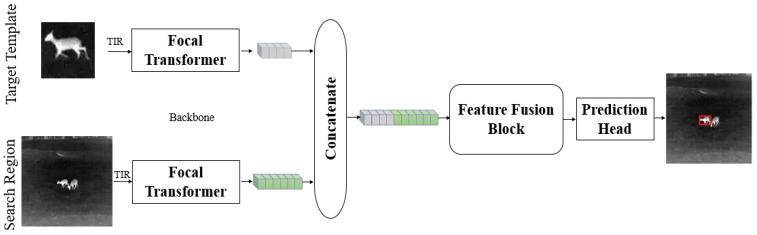
The overall framework of the TIR target tracker.

To solve the current problems in TIR target tracking research, we propose a TIR target tracking framework utilizing the global information, as shown in Figure 2. CNN pays more attention to the local information. In an image, if a large range of dependencies are required to be captured, a large receptive field is needed. Although increasing the size of the convolution kernel can improve the expressiveness of the model, it would also lose the computational and statistical efficiency obtained from the local convolutional structures, while the global contextual information is important for countering interference from similar objects. The remote modeling ability of the Transformer from the attention mechanism has a strong global perception from the beginning of the modeling. In addition, Transformer focuses on the relative relationship between features, not completely dependent on the data itself. The absolute value of the data is no longer as important as in CNN, so its universality and anti-interference are better, and it can better use the synthetic data for neural network training. In the feature extraction process, our TIR target tracking framework utilizes the Focal Transformer network for feature extraction in the template branch and the search branch. During the feature fusion process, a Transformer encoder–decoder is utilized to deeply fuse the extracted features from the template branch and the search branch Stage3. The Transformer decoder connects to the target position prediction head network, which consists of two sub-networks with different structures to form a double prediction head. One fully connected sub-networks is responsible for the classification and prediction of the foreground or background, and the other convolutional sub-network is responsible for the regression prediction of the target bounding box. The entire TIR target tracking framework is simple and efficient. Not only the tracking accuracy and robustness are better than the existing methods, but also the speed of the tracker can reach more than 90FPS.

### 3.1. Feature Extraction Backbone Network

Initially, it was thought that the Transformer network was only effective for natural language processing (NLP) and it was difficult to make achievements in the field of computer vision. Then, Vision-Transformer [19] tried to apply a standard Transformer directly to images, splitting the image into multiple patches and taking the linear embedding sequence of these patches as the input to the Transformer. The image patches are processed in the same way as tokens processed in the NLP domain, and the image classification model is trained in a supervised fashion. Vision-Transformer proved with experiments that the Transformer structure outperforms all methods based on CNN structure on large-scale image classification datasets, and the larger the dataset, the more obvious the advantage Transformer has. Inspired by Vision-Transformer, researchers have begun to try to apply the Transformer structure to other computer vision tasks, such as object detection, segmentation and tracking. TransT [20] and STARK [21] are trackers that have attracted much attention in the field of single target tracking. The basic ideas of them are to use the CNN network to extract the features of templates and search regions, and then use the Transformer structure to fuse the features of the two branches. On the mainstream visible light target tracking evaluation dataset, the performances of TransT and STARK are significantly better than the previous single target tracking framework based on a similarity measure.

**Figure 3:**
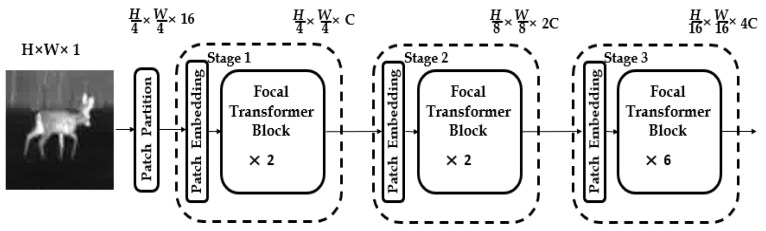
The feature extraction backbone network.

**Figure 4:**
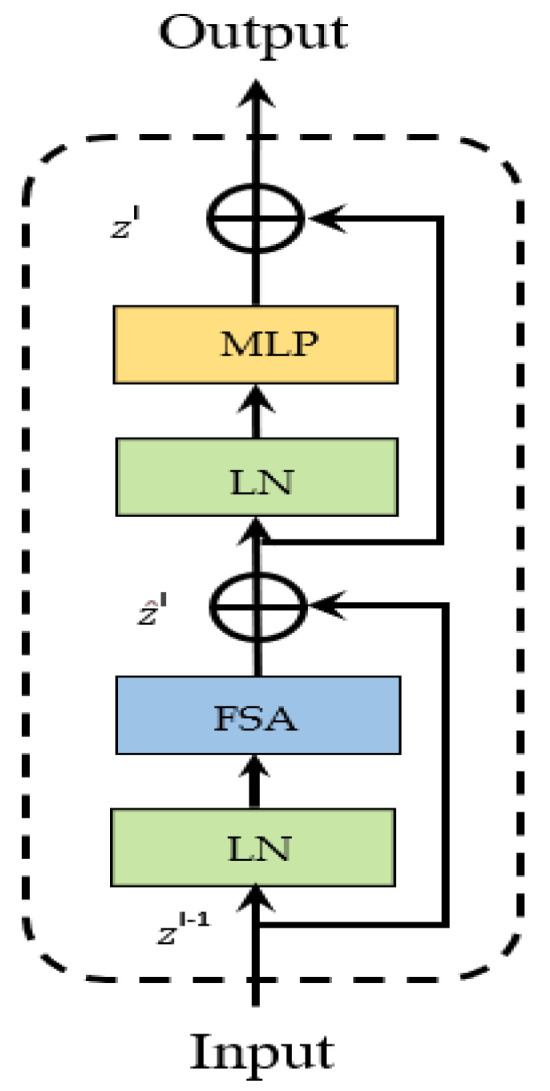
The Focal Transformer block. *FSA* is the focal−based self−attention module. *LN* is the layer normalization layer. MLP is the multi-layer perception network.

There is currently no research using the Transformer structure in the TIR target tracking task. Our method uses the Transformer structure in both the feature extraction and feature fusion processes and utilizes the Focal Transformer as the backbone feature extraction network in the feature extraction process (as shown in Figure 3). The Focal transformer is superior to the original transformer because it utilizes induction bias to extract local image features, such as lines and edges, with a relatively lower cost in global self-attention computation. The feature extraction backbone network of this framework firstly splits the input single-channel TIR image of size H × W into 16 non-overlapping image blocks with the same size (W/4 × H/4) through the Patch Partition module. Then, the tensor of the dimension W/4 × H/4 × 16 is projected to the dimension C with the convolution operation through the Patch Embedding Layer of the image blocks. Additionally, two Focal Transformer blocks (as shown in Figure 4) are utilized to complete the feature extraction in Stage1. The operation of Stage2 and Stage3 is the same as Stage1. After each stage, the size of the feature becomes 1/2 of the previous stage, and the number of feature channels becomes 2 times that of the previous stage. The features generated in the Stage1, Stage2 and Stage3 in the template branch are *z*^1^, *z*^2^ and *z*^3^, respectively; the features generated in the Stage1, Stage2 and Stage3 in the search branch are *x*^1^, *x*^2^ and *x*^3^, respectively. Compared with the traditional Transformer, the Focal Transformer replaces the standard multi-head self-attention module (*MSA*) with a focal-based self-attention module (*FSA*) and keeps other parts unchanged. A layer normalization (*LN*) layer is applied before each *FSA* module and each multilayer perceptron (MLP), and a residual connection is applied after each module. Although batch normalization (BN) layers in CNN can speed up model training and prevent model from overfitting and vanishing gradients, BN is normalized based on the smallest batch, and applying the BN layer directly in the Transformer structure would cause some problems, so our TIR target tracking framework is normalized using *LN* layers. The attention mechanism *FSA* of the Focal Transformer is very similar to the human attention mechanism. When humans look at an object, the center of the retina has the highest resolution, the edge of the retina has the lower resolution, and the clarity gradually decreases from the center to the edge. *FSA* pays attention to the area around the current token in a fine-grained fusion, and to areas far from the current token in a coarse-grained fusion. Local and global attentions can be captured more effectively in this way, and at the same time, the amount of calculation reduces significantly. Assuming that *L* represents the fine-grained level of feature attention in *FSA* (the smaller the subscript of *L*, the more detailed attention of the feature), $s_{w}^{l}$ represents the size of the token sub-window after *l* (*l*∈{1, …, *L*}) aggregation, and $s_{r}^{l}$ represents the number of horizontal and vertical sub-windows of the l-level attention, then the calculation cost of *FSA* attention is shown as Formula (1):

(1) $$O\left(FSA\right)=(L+\sum_{l}{\left(s_{r}^{l}\right)}^{2})\left(M\times N\right)C$$

**Figure 5:**
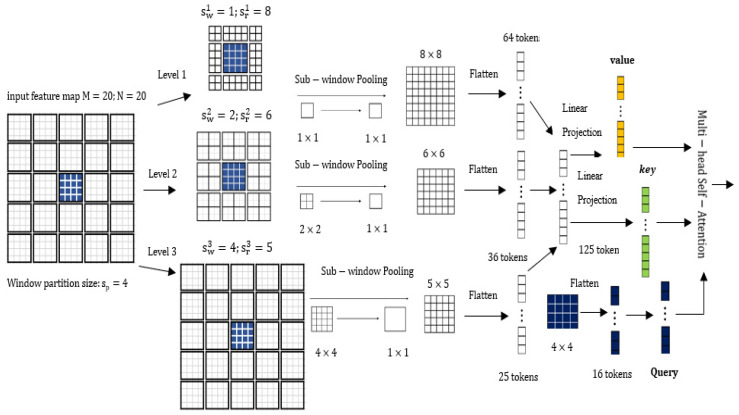
The focus self-attention mechanism in the window level.

Figure 5 shows a typical sample of *FSA*. In Figure 5, each smallest square represents a visual token, and these tokens come from the original feature maps or compressed feature maps. Supposing that there is an input feature map of size 20 × 20, we first divided it into 5 × 5 windows of a size of 4 × 4. Then, we took the middle 4 × 4 blue windows as the query (*Q*), and extracted the tokens around it as its keys (*K*) and values (*V*) at multiple levels of granularity. The first layer extracts the tokens of 8 × 8 closest to the blue window, the sub-window pooling adopts the 1 × 1 to 1 × 1 fusion and the pooled tokens are still 8 × 8. In the second-level attention expansion area, the sub-window pooling adopts 2 × 2 to 1 × 1 method and the pooled tokens become 6 × 6. The third-level attention area covers the entire feature map, the sub-window pooling adopts 4 × 4 to 1 × 1 fusion, and the pooled tokens become 5 × 5. Finally, the tokens of the three levels are flattened and concatenated together to form 125 tokens. These 125 tokens are linearly mapped to *K* and *V*, respectively, and perform multi-head self-attention computation with the tokens (*Q*) in the blue window. *FSA* considers that the visual dependencies between adjacent regions are usually stronger than those between non-adjacent regions, so *FSA* only applies fine-grained self-attention to local regions and applies coarse-grained attention to global regions. Inspired by the Swin Transformer [22], *FSA* incorporates the relative position bias *B* in the attention calculation, as shown in Equation (2):

(2) $$Attention\left(Q,K,V\right)=softmax\left(\frac{QK^{T}}{\sqrt{C}}+B\right)V$$

The value range of the *SoftMax* function is [0, 1], and $K^{T}$ is the transpose matrix of *K*. Dividing by $\sqrt{C}$ is performed to ensure that the gradient is stable during training.

### 3.2. Feature Fusion Network

**Figure 6:**
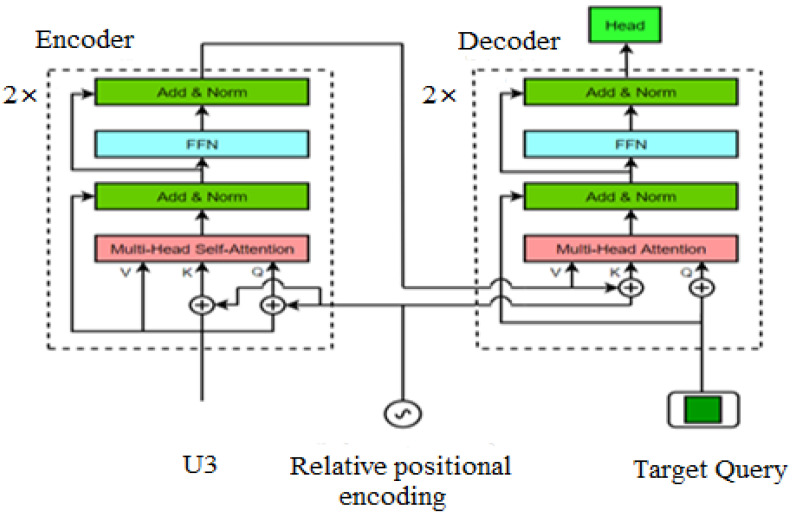
The structure of feature fusion network.

A feature fusion network based on Transformer architecture was utilized in the feature fusion process, as shown in Figure 6. The feature fusion network consists of an encoder and a decoder. The features in the feature extraction stage are processed and input to the encoder. The output of the encoder and the target query are used as the input of the decoder, and the target prediction head network is the output of the decoder. To identify the current position of the processing token, the relative position encoding is the input of the encoder and decoder. Compared with the current mainstream TIR target tracking framework based on CNN cross-correlation operation to generate similarity map, the feature fusion network based on the Transformer architecture outputs features with feature enhancement, which can retain rich semantic information and aggregate global information to establish associations between remote features.

The single-channel TIR images of the template branch and the search branch are extracted by Stage3 to generate features *z*^3^ and *x*^3^, respectively. The numbers of channels of *z*^3^ and *x*^3^ are both 4C. If the feature fusion is performed directly, it will significantly increase the calculation amount of the feature module, so a Bottleneck layer is used to reduce the dimensionality of the features of *z*^3^ and *x*^3^, making the number of channels become d. The features *z^3^* and *x*^3^ after reducing dimensionality are flattened and concatenated to form the new feature *U*^3^ (the number of channels of *U*^3^ is still d), and *U*^3^ is input to the encoder. The encoder consists of two identical layers, each with two sub-layers. The first sub-layer is a multi-head self-attention (*MSA*) network, and the other is a simple fully connected feed-forward network (*FFN*). After the normalization layer, each of these two layers adopts a residual connection. The encoder outputs the feature *U*^5^, and the entire operation process is shown in Equation (3),

(3) $$U^{3}=Conat\left(z^{3},x^{3}\right)\;U^{3^{\prime }}=LN(U^{3}+MSA\left(U^{3}\right))\;U^{4}=LN(U^{3^{\prime }}+FFN\left(U^{3^{\prime }}\right))\;U^{4^{\prime }}=LN(U^{4}+MSA\left(U^{4}\right))\;U^{5}=LN(U^{4^{\prime }}+FFN\left(U^{4^{\prime }}\right))$$

The decoder consists of two identical layers as well, each with two sub-layers. The first sub-layer is a multi-head cross-attention (MCA) network, and the other sub-layer is a simple fully connected feedforward network. The input of the decoder is the output feature of the encoder *U*^5^, and only one target query (*TQ*) is input to the decoder to predict the bounding box of the target. The target query can consider all the locations and search area features on the template to learn a robust representation of the final bounding box. After a series of operations of the encoder and decoder, the feature fusion network finally outputs the feature *U*^7^, which is the original input of the target prediction network (Head). The entire operation process is shown in Equation (4),

(4) $$U^{5^{\prime }}=LN\left(Q+MCA\left(U^{5},TQ\right)\right)\;U^{6}=LN(U^{5^{\prime }}+FFN\left(U^{5^{\prime }}\right))\;U^{6^{\prime }}=LN\left(Q+MCA\left(U^{6},TQ\right)\right)\;U^{7}=LN(U^{6^{\prime }}+FFN\left(U^{6^{\prime }}\right))$$

### 3.3. Prediction Head

**Figure 7:**
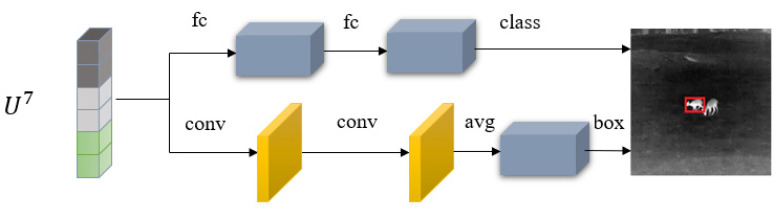
The focus self-attention in the window level. fc is the fully connected layer. conv is the convolutional layer.

The bi-prediction head is composed of two sub-networks with different structures, one fully connected sub-network is responsible for the classification prediction of the foreground or the background and the other convolution sub-network is responsible for the regression prediction of the target bounding box, as shown in Figure 7. Our method does not use a fully connected network, as in the case of TransT, nor only uses a fully convolutional network, as in the case of STARK, but utilizes two different types of networks for target classification and regression, which is inspired by Ref. [23]. Ref. [23] performs detailed experiments to prove that the fully connected head (fc-head) is more sensitive spatially, which is beneficial for foreground or background classification tasks, while the convolution head (conv-head) is more robust in the localization task of the target bounding box regression task. Using a bi-head structure can improve classification robustness and object localization accuracy. The fc-head applies a non-shared transformation (fully connected layer) in different positions of the input feature map and the spatial information is implicit. Therefore, the spatial sensitivity of the fc-head can help to distinguish the complete objects from the partial objects, but it is not robust to determine the offset of the whole object. In contrast, the conv-head uses shared transformations (convolution kernels) in all positions of the input feature map and applies average pooling for aggregation, so it is more suitable for target bounding box regression.

The input features of the two branches are the output features *U*^7^ from the feature fusion network. The feature vectors corresponding to the pixels in the real annotation bounding box of the target are selected as the positive samples, and the feature vectors corresponding to the other pixels are the negative samples. All samples participate in the calculation of the classification loss, but only positive samples are used to calculate the regression loss. To reduce the imbalance between the number of positive samples and negative samples, we reduced the loss from negative samples to 1/10 of the loss from positive samples. The classification loss function adopts the standard binary cross-entropy loss, as shown in Equation (5), where *y_i_* represents the true value label of the *i*-th sample, *y_i_* = 1 represents the foreground and *y_i_* = 0 represents the background. *p_i_* is the probability of the learning model predicting the samples belonging to the foreground.

(5) $${ℒ}_{cls}=-\underset{i}{\sum }[y_{i}\log(p_{i})+(1-y_{i})\log(1-p_{i})]$$

Negative samples do not participate in the calculation of regression loss. The regression loss function adopts GIoU loss [21] (generalized IoU loss), mainly because GIoU is insensitive to the size of the predicted bounding box and the real annotation bounding box of the target. The IoU loss is not only very sensitive to the size of the predicted bounding box and the real bounding box of the target, but also degenerates to the constant 1 and loses the optimization goal when the two objects do not intersect. The regression loss calculation is shown in Equation (6), where *y_i_* represents the true value label of the *i*-th sample, *y_i_* = 1 represents the foreground and *y_i_* = 0 represents the background. *b_i_* represents the predicted bounding box and $\hat{b}$ represents the real bounding box label of the target.

(6) $${ℒ}_{\mathrm{reg}}=\underset{i}{\sum }{𝟙}_{\left\{y_{i}=1\right\}}[{ℒ}_{GIoU}(b_{i},\hat{b})]$$

## 4. Experiments

This section mainly introduces the detailed network structure of our TIR target tracking framework, the generation of the TIR target tracking dataset, the hardware environment of network training and training details of the neural network. At the same time, the effectiveness of the proposed method was verified by the qualitative and quantitative comparison experiments with the current mainstream methods. Finally, rigorous ablation experiments were conducted to verify the impact of each component of the framework on the target tracking performance, and the related principles were analyzed.

### 4.1. Network Structure

The feature extraction backbone network is a Siamese network of three stages, and the template branch and the search branch share the network parameters. The number of Focal Transformer blocks in Stage1, Stage2 and Stage3 are 2, 2 and 6, respectively. Each Focal Transformer block consists of two residual modules, one is composed of the *FSA* layer and the *LN* layer and the other one is composed of the MLP layer and the *LN* layer. The size of the single-channel grayscale image input of the template branch is 112 × 112, and the size of the single-channel grayscale image input of the search branch is 224 × 224. The number of channels in the hidden layer of Stage1 is *C* = 512. The feature fusion network consists of the encoder and decoder with the Transformer structure. The encoder utilizes the multi-head self-attention mechanism. The decoder uses the multi-head cross-attention mechanism and is supplemented with the relative position encoding based on absolute position encoding. The prediction head network is a bi-prediction head network composed of a fully connected sub-network and a convolutional sub-network. The fully connected sub-network has two fully connected layers, which are responsible for the classification of the foreground and the background. The convolutional sub-network has two convolutional layers and one pooling layer, which are responsible for the regression of the target bounding box.

### 4.2. Positional Encoding

**Figure 8:**
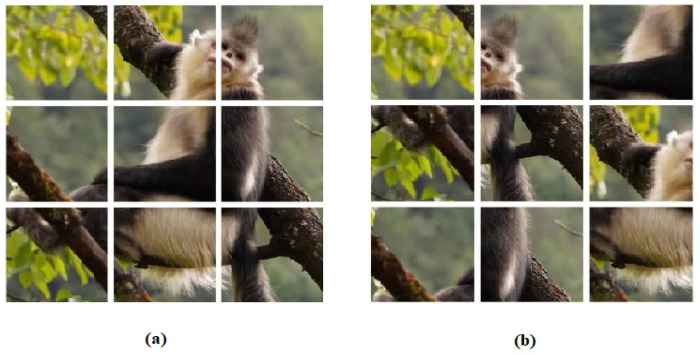
Different sequences in the same image. (**a**) is the sequence of images in normal order, and (**b**) is the sequence of images out of order.

Since self-attention is position-independent, if no positional encoding is added to the image patch, the two sequences in Figure 8a,b look the same to the Transformer. Adding position information before inputting the image patch helps the Transformer to understand the semantic information of elements in the sequence. It was found through experiments that the absolute position encoding method is difficult to learn the semantic difference from different specific positions. Because after the network is trained, all input embeddings are equivalent to adding a fixed value, but the positions and semantic information in different inputs are different. Inspired by Ref. [24], our target tracking framework added relative position encoding to the absolute position encoding. The relative position encoding represents the relative positional relationship between tokens. This relative positional information is added to the self-attention in each layer of the Transformer structure, which makes the model continuously consider the influence of the positional relationship during the learning process. Additionally, it can generalize so as to capture the contained different location information for different input sequences, and thus the Transformer structure can model the semantic information contained in images better.

### 4.3. Network Training

#### 4.3.1. LaSOT-TIR Dataset Generation

**Figure 9:**
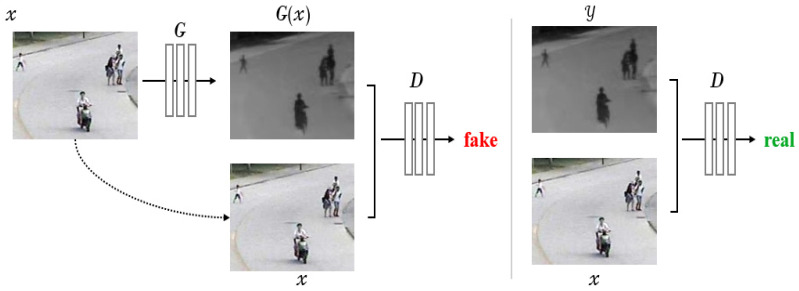
The framework of pix2pix network.

Training the Transformer from scratch requires more data than the CNN network. This is because the CNN can encode the prior knowledge of images, such as translational equivariance, while the Transformer needs to obtain this information from the given data. Although the data scale, scene richness and the number of target types of the LSOTB-TIR dataset have been greatly improved compared with the previous TIR target tracking datasets, the types of challenges in the video sequences are slightly fewer compared with the visible light target tracking datasets, such as GOT10K, LaSOT and TrackingNet. The time and economic costs of establishing a larger and more challenging TIR target tracking dataset are considerable. It is meaningful if high-quality TIR target tracking datasets can be generated by technical methods. The new framework GANs [25] for estimating generative models was proposed by Ian J. Goodfellow et al. in 2014. GANs and their derived models have achieved excellent performances in tasks including image generation, image translation and representation learning. Considering the rigid requirement of the Transformer structure for training data, our method attempts to convert the images in the large-scale long-term target tracking dataset LaSOT of the visible light modality into the TIR modality using pix2pix [26] based on conditional adversarial network (*cGAN*). pix2pix not only learns the mapping from input images to output images, but also learns a loss function to train this mapping, which can apply the same general approach to problems that traditionally require different functions. The input of the generative network *G* of the pix2pix is only *x* (images of the visible light modality), and the output of the neural network after training is *G*(*x*) (images of the TIR modality). Then, *G*(*x*) and *x* are merged together based on the channel dimensions to input to the discriminator *D* and obtain the predicted probability value. The predicted probability value indicates whether the input is a pair of real images. The closer the probability value to 1, the more certain the discriminator *D* is that the input is a pair of real images. The discriminator *D* always tries to identify the “fake” generated by *G* after training, while *G* tries to generate *G*(*x*) that can deceive *D* through training, as shown in Figure 9.

The optimization objective function T* (Equation (7)) of pix2pix consists of two parts. One part is the optimization objective of *cGAN* (Equation (8)), which is the optimized GAN objective optimization function. The other part is the *L*_1_ distance optimization objective (Equation (9)), which is used to constrain the difference between the generated image and the real image. Utilizing the *L*_1_ loss not the *L*_2_ loss is to reduce the blurriness of the generated images.

(7) $$T^{*}=\arg\underset{G}{min}\underset{D}{max}{ℒ}_{cGAN}(G,D)+\lambda {ℒ}_{L1}(G)$$

(8) $$\begin{array}{cc} {ℒ}_{cGAN}\left(G,D\right)= & E_{x,y}\left[\log D\left(x,y\right)\right]+\\ & E_{x,z}\left[\log\left(1-D\left(x,G\left(x,z\right)\right)\right)\right] \end{array}$$

(9) $${ℒ}_{L1}\left(G\right)=E_{x,y,z}\left[∥y-G\left(x,z\right){∥}_{1}\right]$$

λ is the penalty value and *z* represents the random noise. The optimization objective of the discriminator *D* is to make the value of Equation (8) as large as possible, and the optimization objective of the generator *G* is to make the value of log(1 − *D*(*x*,*G*(*x*,*z*)) in Equation (8) as small as possible, which is the meaning of min and max in Equation (7). It should be noted that using the loss function in Equation (8) for network training can be easily to be saturated, which means the discriminator *D* is very strong and the generator *G* is very weak, making *G* hard to train. Therefore, the optimization objective of the generator *G* can be modified from minimization to maximization, that is, log(1 − *D*(*x*,*G*(*x*,*z*))) is modified to be log(*D*(*x*,*G*(*x*,*z*))), and the pix2pix algorithm adopts the modified optimization objective.

**Figure 10:**
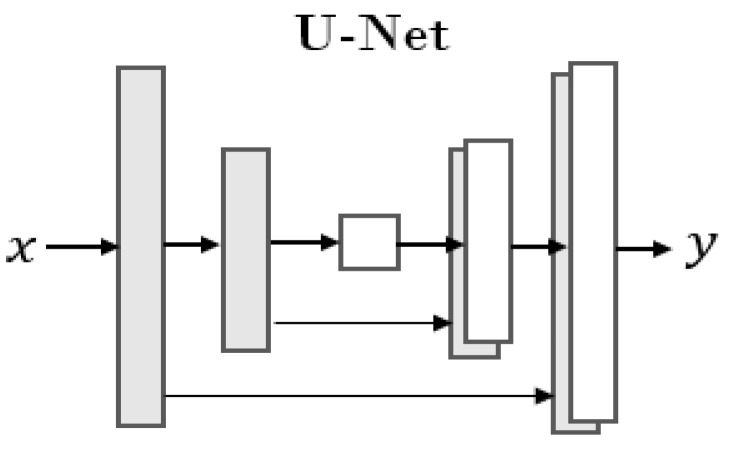
The structure of U-Net network.

In order to fuse features, the generator *G* adopts the network structure U-Net [27], which is widely used in the field of image segmentation, as shown in Figure 10. The discriminator *D* adopts PatchGAN, and it outputs a predicted probability value for each patch of the input image, which means changing from judging whether the input is true or false to judging whether the *N* × *N* area of the input is true or false.

#### 4.3.2. Offline Training

Our TIR target tracking framework was performed on a server with dual Intel Xeon 5218 CPUs and 8 NVIDIA V100 GPUs. The method was implemented based on the deep learning framework PyTorch and Python 3.6. The datasets used for training were the training set of the LSOTB-TIR dataset established by Liu Qiao et al. and all the video sequences of the LaSOT-TIR dataset generated by the pix2pix network, where the LSOTB-TIR dataset contains 1400 TIR image sequences, the training set contains 1280 videos and the evaluation set contains 120 videos. LaSOT-TIR is a long-term target tracking dataset with a total of 1400 TIR image sequences, but this method used all video sequences during training. To maximize the amount of data available for neural network training, data augmentation techniques, including horizontal flipping and brightness jitter, were also utilized during training.

The vanishing gradient makes it difficult to escape from the local optimum and further breaks the optimization stability, so in order not to update the model parameters too quickly initially, our method used AdamW [28] to train the model with a weight decay of 1 × 10^−4^. The initial learning rate of the backbone network was 1 × 10^−5^, and the initial learning rate of other parts was 1 × 10^−4^. The whole training process was 300 epochs, each epoch used 9600 samples and the learning rate was reduced by 10 after 200 epochs. Gradient clipping was used during training to prevent from large gradient misleading optimization process. AdamW added weight decay to the optimizer, decoupling it from the learning rate and playing the role of weight decay.

#### 4.3.3. Online Reasoning

In some sense, our TIR target tracking framework also belongs to the methods based on the Siamese network. So, as in classical methods such as SiamFC [29], the template image was cropped from the first frame of the video sequence, the target object was located in the center of the image and the background area was twice that of the target area. The search area was cropped from the current frame of the tracker, the center of the image was the target center position predicted in the previous frame and the background area of the search area was four times that of the target area. During the online tracking, the prediction head output 1024 prediction boxes with confidence scores, which were then post-processed with a Hanning window penalty. Specifically, a Hanning window with a size of 36 × 36 was applied to the original classification score *S* of the tracker, and the weight value was *λ*. The calculation formula of the final classification score *S_w_* is shown in Equation (10),

(10) $$S_{w}=1-\lambda \times S+\lambda S_{h}$$

where $S_{h}$ is the value of the corresponding position in the Hanning window, and when the value of *λ* is 4.6 in this framework, the tracker achieves the best performance. Based on the Hanning window penalty, the confidence of the feature points far away from the target in the first few frames was penalized, and finally the box with the highest confidence score was selected as the tracking result.

### 4.4. Performance Evaluation

In order to verify the advancedness of our TIR target tracking framework based on efficient global information perception, qualitative and quantitative comparisons were conducted between this proposed model and the current mainstream methods on four TIR target tracking datasets, including VOT2015-TIR [30], VOT2017-TIR [31], PTB-TIR [32] and LSOTB-TIR [2]. From the comparison results, the self-attention mechanism based on Transformer has a strong global perception ability from the beginning of modeling. In addition, the attention mechanism *FSA* of the backbone network Focal Transformer considered the visual dependencies between adjacent areas were usually stronger than the visual dependencies between non-adjacent regions, so the fine-grained self-attention was performed only on local regions and coarse-grained attention was performed on global regions, which makes global information perception more efficient and can improve the speed of the tracker. The comparison results on each dataset are described in detail.

#### 4.4.1. VOT2015-TIR and VOT2017-TIR Dataset

The datasets VOT2015-TIR and VOT2017-TIR are the subsets of the VOT Challenge, where VOT2015-TIR not only contains the evaluation dataset but also provides the corresponding evaluation code platform. It has only 20 TIR video sequences, six target types and a total of 11,000 frames of images. VOT2017-TIR contains 25 image sequences. It removes some simple video sequences in the VOT2015-TIR dataset, adds some challenging video sequences and increases the target types to eight. The quantitative comparison utilizes three metrics: Expected Average Overlap (EAO), Accuracy and Robustness to evaluate the performance of the tracker. It can be found from Table 1 that our method was better than the existing methods in four metrics including EAO, Accuracy, Robustness and Speed. The compared methods include methods based on the manual feature, correlation filtering methods based on the deep feature and matching tracking methods based on deep learning. Among them, on the dataset VOT2015-TIR, the proposed method achieves relative gains of 3.5% and 6.6% in EAO and Accuracy, respectively, compared with MMNet, and Robustness as well as Speed are also significantly better than MMNet. On the dataset VOT2017-TIR, the proposed method achieves relative gains of 5.6% and 8.6% in EAO and Accuracy, respectively, compared with MMNet. The overall performance advantage of our method is more obvious than other methods except for MMNet.

**Figure 11:**
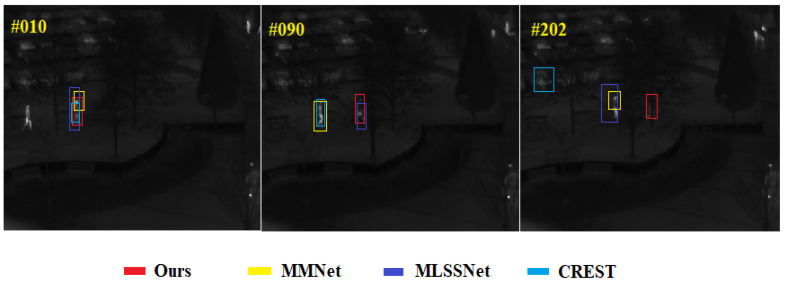
The visualization of tracking results in the sequence trees.

**Figure 12:**
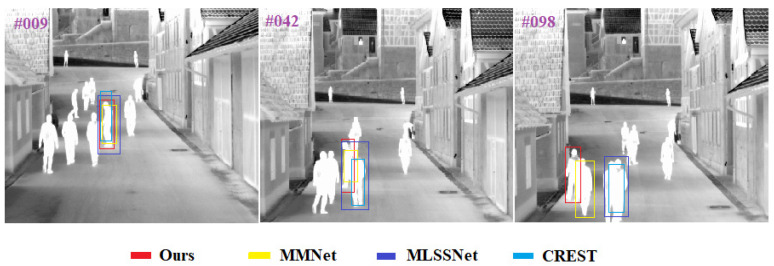
The visualization of tracking results in the sequence saturated.

**Comparison Results of Target Tracking Visualization** In order to more intuitively demonstrate the superior performance of the proposed method, in this section, the results of the proposed method with three methods, including MMNet [13], MLSSNet [33] and CREST [4], that have good performance on the dataset VOT2015-TIR are visualized. Among them, Figure 11 and Figure 12 show the tracking results of four trackers, including our method, on sequence trees and sequence saturated, respectively. These two video sequences contain five challenges, including occlusion, camera motion, dynamic change, motion change and size change. Although occlusion is the main challenge of both the sequence trees and the sequence saturated, the difference is that, in the sequence saturated, the challenges of occlusion and similar interferers occur at the same time, which is obviously more difficult for the tracker. It can be found from Figure 11 that, although none of the four trackers drift at the 10-th frame of the sequence trees, it is clear that our method has the highest accuracy. At the 90-th frame, the target is severely occluded by trees and the MMNet and CREST drift to another person, while our method and MLSSNet do not drift. However, at the 202-th frame, the occlusion by trees of the target is more severe, the target is basically invisible and all three methods except for our method drift. It can be found from Figure 12 that the people in the sequence saturated are dense, the crowd’s movement trajectories are disordered and thermal crossover occurs for multiple times. According to the principle of TIR imaging, it can be known that the infrared radiation values emitted by similar objects with a wavelength of 0.75–13 μm are very similar, the image has no color and the texture features have a high similarity. Combining the above results, it can be found that the sequence saturated has extremely high requirements on the robustness of the tracker. At the 9-th frame, the above four trackers can still track the target, but when the target is occluded and thermal crossover occurs, all three trackers drift except for our tracker, as shown at the 42-th frame and the 98-th frame. The tracking results on the sequence trees and the sequence saturated show that strong global information perception ability is very important for the TIR target tracking tasks, and the method only based on local similarity matching is significantly less robust than the method based on the self-attention mechanism of the Transformer.

#### 4.4.2. PTB-TIR Dataset

**Figure 13:**
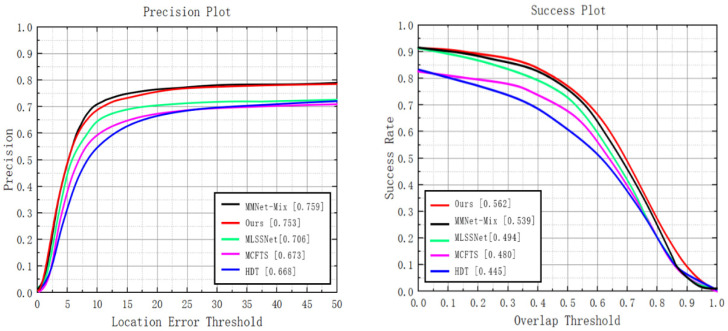
Comparison results with existing methods in the PTB-TIR dataset.

The dataset PTB-TIR is a TIR target tracking dataset for pedestrians with a single target type including only people. There are 60 TIR video sequences in PTB-TIR, the shortest video sequence is 50 frames, the longest video sequence is 1451 frames, and each sequence has nine attribute labels for attribute-based evaluation. The evaluation metrics for PTB-TIR are accuracy and success rate. These two metrics are the results of one run of the tracker, which is usually called the evaluation under OPE. The accuracy was calculated from the average center error, that is, if the distance between the center of the predicted box and the center of the actual bounding box is less than a given threshold (usually 20 pixels or 5 pixels); then, the frame was considered a successful tracking frame, and the accuracy was the average percentage of successful tracking samples from all testing sequences. The success rate was calculated by the average overlap rate. When the overlap rate between the predicted box of the tracker and the actual bounding box was greater than a given threshold, the frame was considered to be a successful tracking frame. The value range of the threshold was from 0 to 1, and the success rate was the ratio of the successful samples of all sequences within the threshold range, that is, the area under the success curve (AUC). As can be seen in Figure 13, our method achieves sub-optimal results with an accuracy of 0.753, which is only 0.8% lower than MMNet (PR = 0.759), which performs the best in the accuracy. However, our method achieves a gain of 4.5% relative to MMNet (SR = 0.562 for the method proposed in this paper; SR = 0.539 for MMNet) in the success rate. Compared to MLSSNet, the third best model on the PTB-TIR dataset, our method achieves a relative increase of 6.7% in accuracy and 13.8% in success rate. MMNet performs better than the proposed method in PR, which denotes that learning the discriminative features and fine-grained correlation features for TIR has a positive influence on improving the tracking accuracy. However, our method performs better than MMNet in SR, which demonstrates that the Transformer structure can model the semantic information contained in images more efficiently, and the excellent ability of global information perception is more important to improve SR.

#### 4.4.3. LSOTB-TIR Dataset

**Figure 14:**
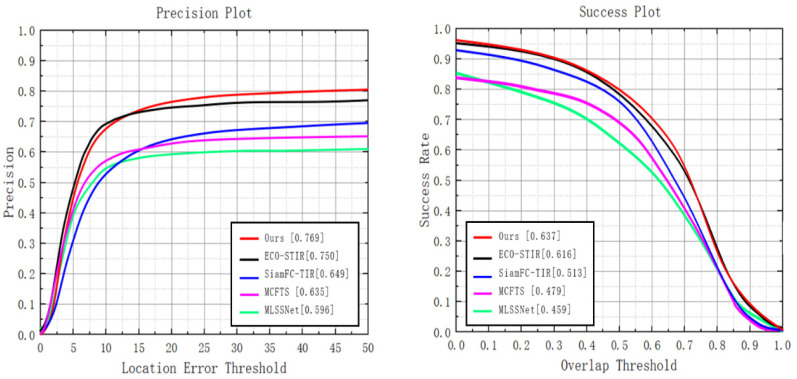
Comparison results with the existing methods in the LSOTB_TIR dataset.

The dataset LSOTB-TIR is the largest known TIR target tracking dataset. LSOTB-TIR not only has the largest variety of video sequences and target types, but also contains more video sequences with challenges of similar interferences, intensity variation and thermal crossover. The videos in the dataset LSOTB-TIR mainly include four scenarios: handheld camera shooting, drone-borne shooting, vehicle-mounted shooting and video surveillance shooting. For the challenge attribute, 12 categories of challenges were defined based on real challenge factors. The dataset LSOTB-TIR contains two subsets, training set and evaluation set, in which the training set contains 1280 video sequences and the evaluation set contains 120 video sequences. LSOTB-TIR also uses center position error (CLE) and overlap ratio (OR) as basic evaluation metrics. Based on these two basic evaluation metrics, the accuracy and success rate under one-pass evaluation were used to measure the overall performance of the tracker. It can be found from Figure 14 that our method achieves the best performance in all metrics on the dataset LSOTB-TIR. Among them, our method achieves the accuracy of 0.769, obtains an increase of 2.5% compared to the second place ECO-STIR and an increase of 18.5% compared to the third place SiamFC-TIR. Our method achieves the success rate of 0.637, obtains an increase of 3.4% compared to the second place ECO-STIR and an increase of 24.2% compared to the third-placed SiamFC-TIR. Our method outperforms other methods, such as ECO-STIR and SiamFC-TIR, in PR and SE. The methods ECO-STIR and SiamFC-FIR utilize the features model of the RGB tracker to represent objects, but these feature models learned in RGB images cannot effectively represent the TIR objects nor take a fine-grained consideration of TIR information, which limits the performance improvement of the tracker.

**Figure 15:**
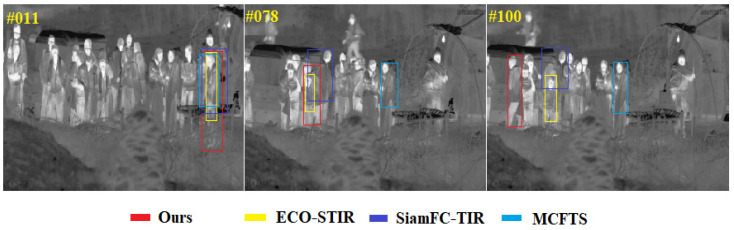
The visualization of tracking results in the sequence person_S_014.

**Figure 16:**
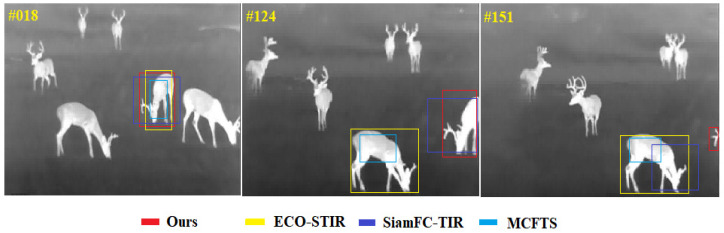
The visualization of tracking results in the sequence deer_H_001.

**Figure 17:**
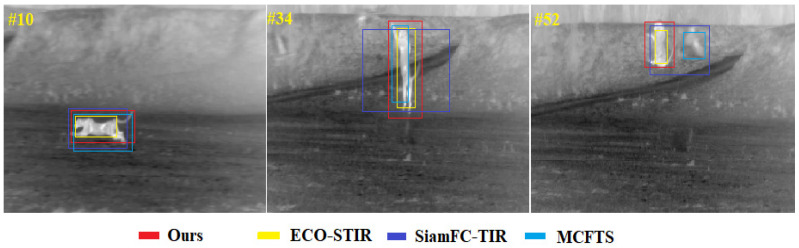
The visualization of tracking results in the sequence leopard_H_001.

In order to more intuitively demonstrate the performance of our method under difficult challenges, including thermal crossover, similar interferences, target reappearance after out-of-view and drastic changes in target aspect ratio, this section compares our methods with the current state-of-the-art methods on the person_S_014 image sequence, the deer_H_001 image sequence as well as the leopard_H_001 image sequence of the evaluation set of LSOTB-TIR, and the results of the comparison are shown. Figure 15 mainly shows the performance of each tracker when thermal crossover and similar object interference occur at the same time. Only the challenge of the thermal crossover or similar interferers is a considerable challenge for TIR target tracking methods, and the current tracking methods cannot cope well with the situation that these two challenges occur frequently at the same time. Our method is the only tracker that does not drift at the 100-th frame. Figure 16 mainly shows the performance of each tracker when the target disappears from (or partially leaves) and reappears in the field of view. This kind of challenge is common in long-term target tracking tasks, mainly evaluating the ability of the tracker to recapture the target. It can be seen from Figure 16 that the target partially leaves the field of view at the 124-th frame. At that time, ECO-STIR and MCFTS drift to the background, and only SiamFC-TIR and our method can still track the target. At the 151-th frame, the shape of the target has fundamentally changed, and it is difficult to discern that it is a deer from its appearance. All methods except ours drifted. Figure 17 mainly shows the performance of each tracker when the shape of the target changes rapidly during the tracking process. The sequence leopard_H_001 shows the process of the cheetah jumping up and trying to capture the birds in the air after quickly running. At the 10-th frame, the four trackers in the comparison can track the target well, but the accuracy of the predicted box of ECO-STIR is significantly lower than the other three trackers, and it does not include the tail of the cheetah in the predicted box. However, at the 34-th frame, SiamFC-TIR performs the worst, which shows that SiamFC-TIR cannot adapt well to the drastic changes in the target aspect ratio. At the 52-th frame, MCFTS drifts to the bird, and only our method can accurately track the cheetah.

#### 4.4.4. Ablation Experiments

To demonstrate the effectiveness of each part of our TIR target tracking framework, this section conducts the ablation experiments on the VOT2017-TIR dataset. The ablation analysis is mainly performed from two aspects: the feature extraction backbone network and the prediction head network. After the ablation experiments, it can be seen that the feature extraction backbone network has a greater impact on the performance of the tracker than the prediction head network.

**Feature Extraction Backbone Network** The feature extraction backbone network is the basis of the tracking framework. The previous tracking frameworks utilize CNN networks as the feature extraction backbone network, so researchers have only focused on the number of parameters and layer types of the feature extraction backbone network. Our method used the Transformer structure for the feature extraction of template images and searched images without applying the widely used ResNet-50 as the feature extraction backbone network. Specifically, our framework utilized the Focal Transformer as the feature extraction backbone network. It can be seen from Table 2 that, when the other modules are the same, using the Focal Transformer as the feature extraction backbone network can improve the EAO of the tracker by up to 4%, the accuracy by up to 4.5% and the robustness by up to 8.4% compared with ResNet-50 as the feature extraction backbone. The self-attention mechanism of the Focal Transformer has a strong global perception ability from the beginning of modeling. Additionally, the attention mechanism *FSA* considers that the visual dependencies between adjacent regions are usually stronger than those between non-adjacent regions. Therefore, only fine-grained self-attention is performed on local regions, while coarse-grained attention is performed on global regions, which makes global information perception more efficient.

**Prediction Head Network** Different from the two tracking frameworks based on the Transformer structure TransT and STARK, our method consists of two sub-networks with different structures to form a bi-prediction head, where one fully connected (FC) sub-network is responsible for the classification prediction of the foreground or background and the other convolutional (CNN) sub-network is responsible for regression prediction of the target bounding box. This section demonstrates the rationality of heterogeneous bi-prediction heads with the ablation experiments. It can be seen from Table 2 that, when the backbone network Focal Transformer and the heterogeneous bi-prediction head (FC + CNN) are used, the EAO of the tracker is 0.338, which achieves an increase of 0.9% compared with the dual FC prediction head and an increase of 2.1% compared with the dual CNN prediction head. When ResNet-50 is utilized as the backbone network, the EAO of the tracker is 0.325 with the heterogeneous bi-prediction heads, which achieves a 0.6% increase compared with the dual FC prediction heads and a 1.9% increase compared with the dual CNN prediction heads. In addition, from the accuracy and robustness metrics in Table 2, it can be found that no matter which feature extraction backbone network is utilized, the performance of the heterogeneous bi-prediction head tracker is the best. Additionally, using dual FC prediction heads is better than dual CNN prediction heads in robustness, but using dual CNN prediction heads is better in accuracy. Ablation experiments show that the fully connected head is more spatially sensitive, which is beneficial for foreground or background classification tasks, while the convolutional head is more robust in the task of target box regression. Utilizing a dual-head structure can improve the overall performance of the tracker.

## 5. Conclusions

The Focal Transformer was used in the feature extraction process to improve the efficiency of remote information modeling. The attention mechanism *FSA* utilized in the Focal Transformer was a new self-attention mechanism that combines fine-grained local interaction and coarse-grained global interaction. In this new mechanism, each token solves its nearest surrounding tokens at a fine-grained level and solves distant tokens at a coarse-grained level, thus effectively capturing both short-term and long-term visual dependencies. In the feature fusion process, relative position encoding is supplemented with the standard Transformer structure, which allows the model to continuously consider the influence of positional relationships during the learning process and can generalize to capture contained different positional information for different input sequences, making the Transformer structure a better model of the semantic information contained in images. In the object prediction process, utilizing two sub-networks with different structures to form the bi-prediction head helps to improve the overall performance of the tracker. Training on the LaSOT-TIR dataset can improve the ability of the tracker to withstand the typical challenges of the long-term object tracking. The qualitative and quantitative comparison with the current mainstream methods on TIR target tracking datasets, such as VOT2015-TIR, VOT2017-TIR, PTB-TIR and LSOTB-TIR, proves that our method is better than the existing methods.

## Figures and Tables

**Table 1:** Performance comparison with other existing tracking methods on VOT2015-TIR and VOT2017-TIR.

Tracking Method	VOT2015-TIR			VOT2017-TIR			Speed
	EAO↑	Acc↑	Rob↓	EAO↑	Acc↑	Rob↓	FPS
Ours	0.356	0.65	2.01	0.338	0.63	2.56	90.0
MMNet	0.344	0.61	2.09	0.320	0.58	2.91	18.9
MLSSNet	0.329	0.57	2.42	0.286	0.56	3.11	18.0
CREST	0.258	0.62	3.11	0.252	0.59	3.26	0.6
TADT	0.234	0.61	3.33	0.262	0.60	3.18	42.7
SRDCF	0.225	0.62	3.06	0.197	0.59	3.84	12.3
Siamese-FC	0.219	0.60	4.10	0.225	0.57	4.29	66.9

**Table 2:** The results of the ablation experiments in VOT2017-TIR.

Backbone Network	Prediction Head Network	VOT2017-TIR		
		EAO↑	Acc↑	Rob↓
Focal Transformer	FC (cla.) + CNN (reg.)	0.338	0.63	2.56
Focal Transformer	FC (cla.) + FC (reg.)	0.335	0.58	2.59
Focal Transformer	CNN (cla.) + CNN (reg.)	0.331	0.60	2.61
ResNet-50	FC (cla.) + CNN (reg.)	0.325	0.55	2.76
ResNet-50	FC (cla.) + FC (reg.)	0.323	0.51	2.79
ResNet-50	CNN (cla.) + CNN (reg.)	0.319	0.53	2.83
